# MicroRNAs’ Therapeutic Potential in Multiple Human Diseases

**DOI:** 10.3390/ijms27209371

**Published:** 2026-10-21

**Authors:** Hua Zhang, Zhengxu Cai

**Affiliations:** 1 Department of Neurology, The First Affiliated Hospital of Dalian Medical University, Dalian 116044, China; 17866525908@163.com; 2 Research Center, Dalian Medical University, Dalian 116044, China

**Keywords:** microRNAs, central nervous system diseases, metabolic diseases, tumors, cardiovascular diseases

## Abstract

MicroRNAs (miRNAs) are a class of short single-stranded non-coding RNAs widely present in multicellular eukaryotes, including animals, plants, and fungi. Typically, approximately 22 nucleotides in length (with some ranging from 22 to 25 nucleotides), miRNAs are predominantly localized to non-coding genomic regions and exhibit high evolutionary conservation. As key post-transcriptional modulators of gene expression, miRNAs are non-protein-coding molecules. They bind complementarily to the 3′UTR of target mRNAs. This interaction triggers mRNA breakdown or inhibits protein translation. In this way, miRNAs regulate nearly 30% of cellular protein expression. MiRNA biogenesis is tightly regulated. Abnormal miRNA expression alters disease-related genes and signaling pathways. It further drives the onset, development, and pathological processes of various human diseases. This review outlines recent progress in research on miRNA functions across multiple illnesses. These illnesses cover neurological, metabolic, tumor, and cardiovascular disorders.

## 1. Introduction

MicroRNAs, or miRNAs for short, are short non-coding RNAs. They exist in nearly all multicellular eukaryotes and carry strong evolutionary conservation. These molecules take charge of gene regulation after transcription. miRNAs mature through a series of strictly coordinated biochemical reactions. Once mature, they bind to the RNA-induced silencing complex (RISC). The complex then binds the 3′ untranslated regions (3′UTRs) of target mRNAs. This binding triggers mRNA degradation or blocks protein translation. Through this mechanism, miRNAs control around 30% of proteins produced in human cells. Plenty of preclinical studies support the vital roles of miRNAs in disease. Abnormal miRNA expression disrupts many core biological pathways. These pathways regulate inflammation, cell proliferation, apoptosis, tissue fibrosis, and neural remodeling. Such molecular disorders eventually drive the initiation and progression of diverse human diseases. Most existing miRNA reviews focus on only one organ system. Some discuss neurological injuries alone, while others only cover cancers or cardiovascular diseases. Few reviews integrate evidence covering neurological metabolic malignant cardiovascular and renal diseases simultaneously.

We conducted a systematic literature search for this review. We retrieved articles from three databases. The databases include PubMed Web of Science and Scopus. Our search time window spanned from January 2010 to December 2024. Core search keywords included microRNA and miRNA non-coding RNA, together with epilepsy, stroke, Parkinson’s disease, Alzheimer’s disease, multiple sclerosis, diabetes, cancer, heart failure, myocardial infarction, atherosclerosis, and kidney injury. We set clear literature inclusion criteria. Original research articles and high-quality review papers were included. Studies needed to report miRNA-related functional experiments or clinical sample detection results. We excluded conference abstracts case reports and non-English publications.

We selected disease categories according to the following basis. We prioritized diseases with abundant preclinical miRNA functional evidence. We focused on disorders with clear translational prospects for miRNA-based diagnosis and treatment. We did not cover viral diseases and autoimmune diseases. This choice was made because existing miRNA research for these conditions remains fragmented. Many mechanistic conclusions lack sufficient in vivo verification. We acknowledge this limitation. Related research directions can be explored in future specialized reviews.

We organized content according to the following logic. We first introduce clinical and pathological features for each disease. We then elaborate on the regulatory roles of dysregulated miRNAs under such disease backgrounds. This review starts with the classic biogenesis process of miRNAs and explains their core regulatory mechanisms. We collect recent findings from preclinical studies. Based on these data, we analyze how dysregulated miRNAs participate in pathological changes of various diseases. These diseases include epilepsy, stroke, neurodegenerative disorders, diabetes, multiple cancers, heart failure, myocardial infarction, atherosclerosis, and kidney injury. We also sort out tissue-specific functional miRNAs. We list their downstream target genes and corresponding signaling pathways verified in different animal disease models. In the final section we evaluate the potential of miRNAs to act as diagnostic biomarkers and therapeutic targets. We summarize major obstacles restricting clinical transformation. Key obstacles contain low in vivo delivery efficiency and off-target adverse effects. We also discuss technical limitations for miRNA detection. We cover exosome-based delivery strategies for miRNA therapy. We put forward practical research directions to promote clinical translation of miRNA-based diagnosis and treatment.

## 2. Biogenesis and Regulatory Mechanisms of miRNAs

**Figure 1:**
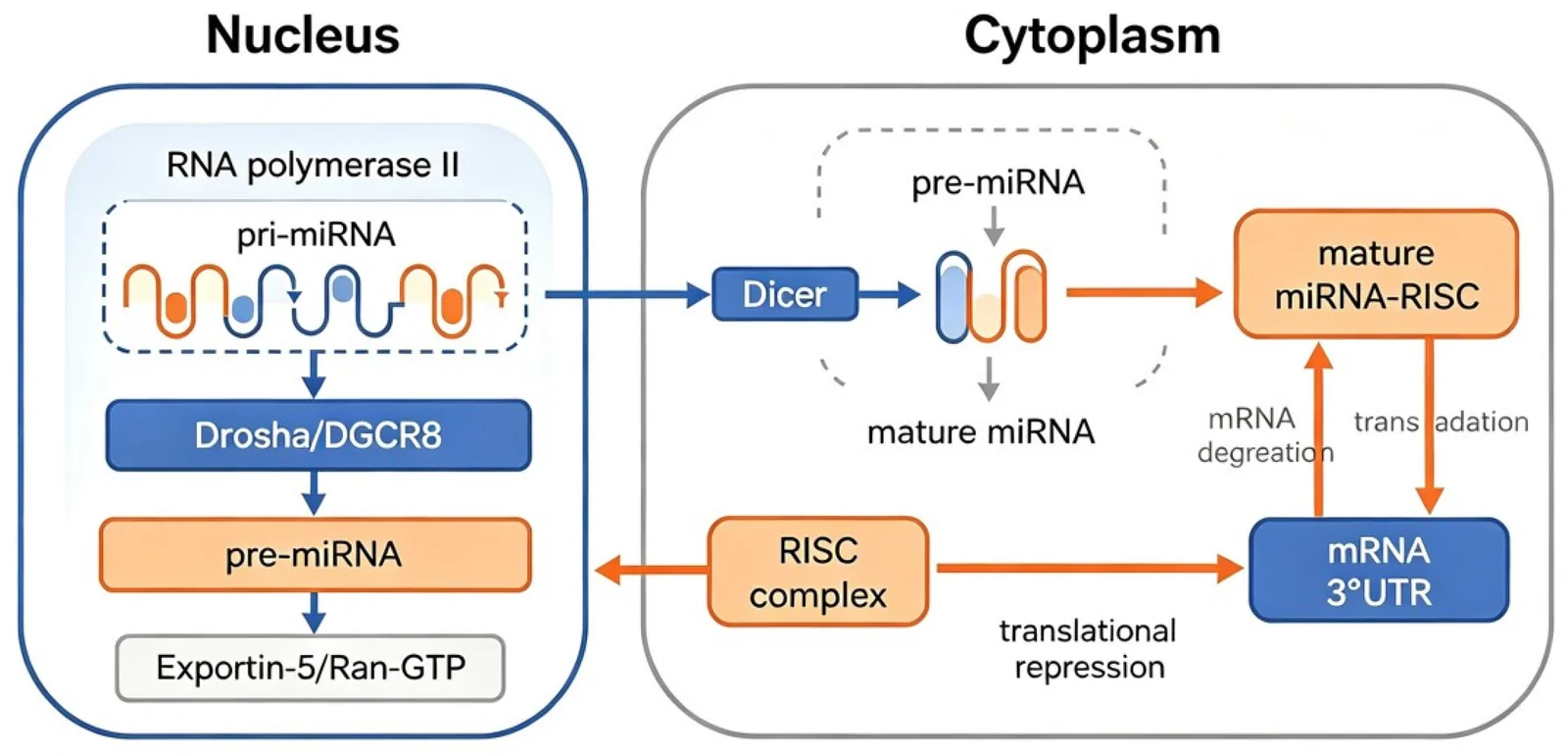
Biogenesis and regulatory mechanism of microRNAs. This diagram illustrates nuclear processing and cytoplasmic maturation steps for miRNA. It shows how mature miRNA-RISC complex suppresses target mRNA through degradation or translational repression.

MiRNA biosynthesis undergoes multiple rigorous steps, mainly consisting of nuclear processing and cytoplasmic maturation, as shown in Figure 1. Most miRNA promoters are recognized by RNA polymerase II, yielding an initial transcript termed primary miRNA (pri-miRNA) of approximately 1000 base pairs (bp). In the cell nucleus, Drosha, an RNase III endonuclease, binds DGCR8 to form a functional complex. This complex identifies pri-miRNAs through double-stranded RNA-binding domains. It cuts 11 base pairs downstream to produce stem–loop pre-miRNAs of 60–100 bp. These precursors possess 5′ phosphate ends and special terminal overhangs. Exportin-5 then identifies the 3′ dinucleotide overhang of pre-miRNAs. Together with Ran-GTP, it transports these precursors from the nucleus to the cytoplasm. In the cytoplasm, the Dicer enzyme recognizes pre-miRNAs and cleaves the duplex regions flanking the stem–loop to produce a 19–23-nucleotide double-stranded RNA (structurally analogous to small interfering RNAs). One strand gets degraded. The other strand develops into a mature functional miRNA. This mature miRNA is integrated into the RISC complex. It binds the 3′UTR of target mRNAs to regulate gene expression [1,2].

Collectively, the multi-step biogenesis cascade tightly controls the abundance and activity of mature miRNAs. Any disturbance in Drosha/DGCR8 cleavage, cytoplasmic Dicer processing, or RISC assembly alters miRNA bioavailability, thereby disrupting the post-transcriptional repression of hundreds of disease-relevant target transcripts. Given that miRNA-mediated signaling governs core cellular events, including inflammation, apoptosis, proliferation and fibrosis, dysregulation of this maturation machinery directly participates in the initiation and progression of diverse human disorders. In the following sections, we systematically dissect aberrant miRNA expressions and their pathogenic regulatory networks across neurological, metabolic, malignant, cardiovascular and renal diseases.

## 3. miRNAs and Central Nervous System Diseases

### 3.1. miRNAs and Epilepsy

**Figure 2:**
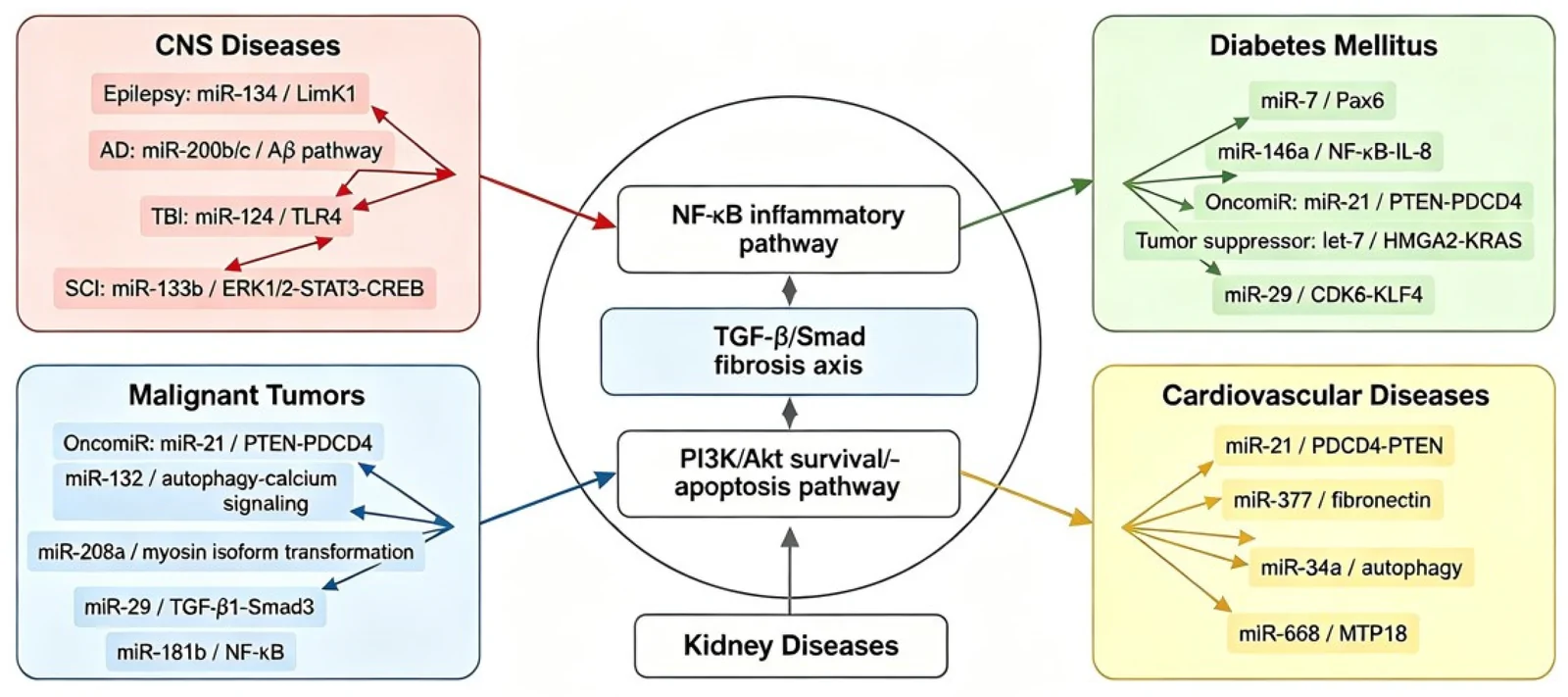
Core shared signaling pathways regulated by representative miRNAs across different disease systems. This schematic displays key miRNAs from central nervous system diseases, diabetes, malignant tumors, cardiovascular diseases, and kidney diseases. It links these miRNAs to shared downstream signaling cascades, including NF-κB inflammatory pathway, TGF-β/Smad fibrosis axis, and PI3K/Akt survival–apoptosis pathway.

As shown in Figure 2, epilepsy is a common chronic central nervous system neurological disease. Its pathogenesis is closely associated with abnormal neuronal excitability and electrical discharges. We first describe core disease characteristics for epilepsy. Epileptogenesis refers to the progressive pathological process that drives epilepsy formation. It covers brain injury latency period and development of spontaneous recurrent seizures. In contrast, acute seizure response describes transient neuronal hyperexcitability during single seizure episodes. Distinct sets of miRNAs participate in these two biological processes.

Growing evidence has proven that miRNAs are involved in the pathological modulation of epilepsy. A large number of dysregulated miRNAs have been reported in the epilepsy research. Many studies focus on circulating miRNAs from plasma serum and cerebrospinal fluid. These circulating molecules show promising biomarker values for epilepsy and drug-resistant epilepsy. Earlier animal studies demonstrated that the intracortical administration of miR-134 antisense oligonucleotides markedly attenuated epileptic seizures in a kainic acid-induced mouse model of epilepsy. This effect is thought to be mediated via regulation of the dendritic spine regulator LimK1, and the antiepileptic activity can persist for more than 2 months [3,4]. This evidence originates from in vivo mouse model experiments. Recent investigations have revealed that the knockdown of miR-135a in the amygdala effectively alleviates epileptic seizures in kainic acid-induced epilepsy models [5]. This result was obtained from rodent animal experiments. In both kainic acid- and piracetam-induced epilepsy models, the inhibition of miR-324-5p reduces seizure frequency by modulating Kv4 channels [6,7]. These conclusions are supported by animal model data. Furthermore, in piracetam-and pentylenetetrazol-induced epilepsy models, direct administration of miR-137 mimics enhances the release of inhibitory neurotransmitters and prolongs seizure latency in mice [8]. These observations derive from rodent in vivo studies.

Multiple other miRNAs are involved in epileptogenesis or acute seizure modulation. For example miR-128 participates in epileptogenesis by regulating neuronal apoptosis and calcium homeostasis in rodent epilepsy models. Circulating miR-146a in serum shows altered expression in clinical patients with drug-resistant temporal lobe epilepsy. Some studies report inconsistent expression changes for certain circulating miRNAs across different patient cohorts. Such contradictions may come from differences in sample collection time patient age and etiology. These unresolved points need further large-sample clinical validation.

Multiple miRNAs participate in either chronic epileptogenesis or acute seizure response. Preclinical animal experiments support the anti-seizure function of several miRNA inhibitors or mimics. Circulating miRNAs serve as potential non-invasive biomarkers. But existing clinical evidence still contains conflicting results. More human patient cohort studies are required to verify their practical application value.

### 3.2. miRNAs and Stroke

Stroke, encompassing cerebral infarction and intracerebral hemorrhage, is a leading cause of neurological impairment. Stroke pathological process covers acute neuronal death inflammatory activation and subsequent neural repair. MiRNAs exert dual regulatory effects on injury progression and functional recovery after stroke. miRNAs play pivotal roles in the pathological processes of stroke and in the restoration of neurological function. Previous studies have shown that, in cerebral infarction, the miR-17-92 cluster regulates the PI3K/Akt/mTOR pathway to promote neuronal axonal and myelin sheath remodeling, thereby facilitating neurological recovery after cerebral infarction. This regulatory mechanism is validated in rat middle cerebral artery occlusion animal models. Additionally, miR-126 promotes angiogenesis and neurogenesis by upregulating VEGFR2, accelerating neurological repair following cerebral infarction [9,10]. This result comes from endothelial progenitor cell experiments and rodent stroke models.

Many additional miRNAs participate in stroke-related brain injury. MiR-155 aggravates neuroinflammation after ischemic stroke in mouse models. MiR-497 promotes neuronal apoptosis after cerebral ischemia reperfusion injury. Some human clinical studies detect expression shifts of circulating miR-124 and miR-9 in plasma samples from patients with stroke. Several independent cohorts report inconsistent fold change for these circulating markers. Such discrepancies may relate to sample collection time points, stroke subtype, and patient comorbidities.

A panel of miRNAs modulates neuronal survival inflammation and vascular regeneration after stroke. Most functional conclusions are derived from preclinical cell and animal experiments. Circulating miRNAs show potential diagnostic value for stroke. Heterogeneous results exist among different human clinical cohorts. Standardized multi-center patient studies are needed to confirm their clinical utility.

### 3.3. miRNAs and Parkinson’s Disease

Parkinson’s disease belongs to neurodegenerative diseases. Its main feature is the death and damage of dopaminergic neurons in the substantia nigra compacta. Altered miRNA levels are strongly related to disease occurrence and development. MiR-155 acts as a typical pro-inflammatory miRNA. It promotes microglia-mediated neuroinflammation in Parkinson’s disease. The inhibition of miR-155 can produce neuroprotective outcomes. Early studies obtained conflicting observations in different model systems. One study reported that miR-155 suppresses microglial activation and reduces loss of tyrosine hydroxylase (TH)-positive neurons in Parkinson’s disease mouse models [11]. This observation contradicts most other published studies. We note this inconsistency. More model and human tissue evidence is required to clarify the exact function of miR-155 in Parkinson’s disease. Another study reported that the downregulation of miR-205 elevates LRRK2 mRNA expression and increases susceptibility to Parkinson’s disease [11]. This result was validated in human cell experiments and post-mortem human brain tissue samples.

More disease-relevant miRNAs have been documented for Parkinson’s disease. MiR-34b/c downregulates alpha-synuclein expression in cell models. Circulating miR-132 in cerebrospinal fluid displays expression changes in Parkinson’s patient cohorts. A small number of studies report opposite expression trends for these markers across patient subgroups.

MiRNAs participate in alpha-synuclein pathology microglial inflammation and neuronal survival in Parkinson’s disease. Some miRNA functional conclusions remain controversial across research reports. Evidence from human post-mortem brain tissue and patient biofluids should be given higher priority in future research.

### 3.4. miRNAs and Alzheimer’s Disease

As shown in Figure 2, Alzheimer’s disease (AD) is a frequent senile neurodegenerative dementia. Its key pathological features include Aβ aggregation and excessive tau hyperphosphorylation. Multiple in vivo AD animal models have been applied to explore miRNA functions. These models include 5×FAD transgenic mice, APPswe/PS1dE9 mice, and SAMP8 mice. MiRNAs modulate related target genes and thus take part in AD pathological processes. Previous studies demonstrated that miR-200b/c mimics prevent memory loss and learning deficits in AD mouse models [12]. This evidence is obtained from a 5×FAD AD mouse model. MiR-188-5p counteracts Aβ42-induced synaptic toxicity and cognitive impairment [13]. The experiment was conducted using 5×FAD mice. MiR-132, miR-219-5p, and miR-483-5p reduce tau phosphorylation and ameliorate memory deficits in AD mice [14]. These findings are supported by transgenic AD animal models. Moreover, miR-31-5p downregulates β-secretase 1 (BACE1) expression, mitigating amyloid deposition and cognitive decline [15]. This result comes from an APP/PS1 AD mouse model. miR-299-5p and miR-214-3p reduce neuronal apoptosis and improve cognitive function in AD mice [16,17]. Observations are from APPswe/PS1dE9 and SAMP8 mouse models. miR-326 inhibits neuronal apoptosis by targeting Bax, Bcl-2, and caspase-3 through apoptotic signaling cascades, thereby enhancing cognitive performance [18]. This conclusion derives from cell experiments and AD mouse models. The upregulation of miR-16 reduces tau phosphorylation and neuronal dendritic damage, improving memory function [18]. MiR-34c-5p is associated with learning impairment and is highly expressed in patients with AD [19]. This result is supported by human patient blood sample detection.

Several miRNAs show inconsistent results between animal models and human patient samples. For example some miRNAs produce protective effects in transgenic mice. Their expression changes cannot be replicated in the peripheral blood of patients with AD. Such a gap reminds researchers to cautiously translate preclinical findings to clinical practice.

MiRNAs can regulate Aβ generation tau phosphorylation neuronal apoptosis and synaptic function in multiple AD animal models. Limited consistency exists between animal-derived results and human patient biofluid data. Additional human brain tissue and large patient cohort studies are needed.

### 3.5. miRNAs and Multiple Sclerosis

Multiple sclerosis (MS) is a chronic inflammatory CNS demyelinating illness, and its development is tightly linked to abnormal immune cell differentiation. miRNAs are implicated in the regulatory networks underlying MS pathogenesis. Studies have shown that miR-326 promotes the differentiation and maturation of T helper 17 (Th17) cells, thereby contributing to MS pathogenesis [20]. This evidence comes from EAE mouse model and human immune cell experiments. Another investigation revealed that let-7i inhibits regulatory T cell differentiation and participates in the pathological regulation of MS [21]. This result was observed in human T cell culture systems.

MiR-155 represents another key mediator of central nervous system inflammation during MS progression. Increased miR-155 expression amplifies inflammatory responses within the central nervous system in EAE animal models through driving pro-inflammatory macrophage and microglial activation. Genetic ablation of miR-155 can mitigate demyelination and reduce clinical disease severity in preclinical MS animal models. Many other miRNAs participate in MS immune disturbance. Circulating serum miR-21 exhibits altered expression profiles among multiple sclerosis patient cohorts. Its expression level may correlate with inflammatory burden in patients with MS. Several research teams report conflicting expression patterns for some circulating miRNAs. These divergent findings may stem from differences in patient disease activity and treatment status.

MiRNAs modulate Th17 Treg and other immune cell functions in multiple sclerosis. Current functional evidence mainly comes from EAE animal models and in vitro human immune cell assays. Clinical sample results carry certain inconsistency. Further research should connect miRNA expression profiles with actual disease phenotype and clinical activity.

### 3.6. miRNAs and Traumatic Brain Injury (TBI)

As shown in Figure 2, TBI represents a common form of central nervous system trauma. miRNAs modulate microglial polarization, neuronal autophagy, and inflammatory responses, thereby influencing post-TBI functional recovery. Research has verified related regulatory mechanisms after TBI.MiR-124 restrains TLR4 to induce microglial M2 polarization and aid TBI recovery [22]. This conclusion comes from in vitro microglia experiments and TBI mouse models. MiR-873a-5p inhibits NF-κB and facilitates M2 polarization to restore neurological function [23]. Evidence originates from astrocyte–microglia co-culture and rodent TBI models. MiR-124-3p suppresses neuronal autophagy and exerts neuroprotective roles post-TBI [22]. This result is validated in mouse TBI models. The miR-17-92 cluster boosts neurogenesis and angiogenesis. It also relieves neuroinflammation and neuronal injury [9]. This finding is supported by bone marrow mesenchymal stem cell experiments and rat TBI models.

MiRNAs control microglia phenotype neuronal autophagy and neurorepair after traumatic brain injury. Most functional data are obtained from cell culture and rodent trauma models.

### 3.7. miRNAs and Spinal Cord Injury

As shown in Figure 2, spinal cord injury refers to severe central nervous system trauma, which frequently causes sensory and motor impairments. miRNAs play important roles in neural repair following spinal cord injury. Research has confirmed multiple miRNA functions in spinal cord injury. MiR-133b activates ERK1/2, STAT3, and CREB pathways to accelerate neuronal axon regeneration, providing a promising treatment direction [24]. This result comes from mesenchymal stem cell exosome experiments and rat spinal cord injury models. MiR-29b modulates NF200, GAP-43, and GFAP to regulate neuronal regeneration [25]. This observation derives from rat spinal cord injury models. MiR-544 curbs pro-inflammatory cytokines, eases post-traumatic inflammation, and facilitates tissue repair [26]. This evidence is obtained from in vivo rat experiments. In addition, the miR-199a-3p/145-5p axis boosts axonal growth and inhibits neuronal apoptosis. It ultimately promotes functional recovery in spinal cord injury rats [27]. This conclusion comes from umbilical mesenchymal stem cell-derived exosome research and rat injury models.

MiRNAs regulate axonal regeneration glial scar formation and local inflammation after spinal cord injury. Preclinical animal studies support their repair promoting potential.

## 4. miRNAs and Diabetes

Diabetes mellitus is a metabolic disease mainly marked by a disturbed glucose balance. Its main causes include abnormal insulin production, secretion, and glucose metabolism disorders. Two major clinical subtypes include type 1 diabetes and type 2 diabetes. Type 1 diabetes arises from the autoimmune destruction of pancreatic beta-cells. Type 2 diabetes accounts for most clinical cases. It is mainly driven by insulin resistance in adipose tissue liver and skeletal muscle followed by relative beta-cell dysfunction. We describe miRNA evidence for both subtypes in this section.

MiRNAs take part in the pathological regulation of diabetes, as shown in Figure 2 and Table 1. Evidence reveals that elevated miR-130a, miR-130b, and miR-152 target GCK and PDHA1 to regulate glycolysis, thus lowering insulin synthesis and release [28]. This finding is validated in pancreatic beta-cell line experiments. Previous studies indicated that miR-7 downregulates Pax6 and blocks insulin gene transcription, which accelerates the progression of diabetes [29,30]. This evidence comes from human and rodent pancreatic islet cell experiments. Glucose induces miR-30d expression. It stimulates insulin gene transcription [31]. This result is observed in pancreatic beta-cell culture. Glucose upregulates miR-124a expression. It also enhances insulin secretion [32,33]. This conclusion is supported by human type 2 diabetic islet samples and cell experiments.

Upregulated miR-181a targets PTEN during early islet autoimmunity. It activates PI3K-NFAT5 signaling and elevates CD28 expression. This drives excessive T cell activation and impairs Treg differentiation. miR-181a inhibition restores Treg induction and alleviates autoimmunity.

Such mechanisms have been validated in NOD mouse models of type 1 diabetes [34,35]. These results are from type 1 diabetes NOD mouse in vivo experiments. MiR-142-3p is markedly upregulated under islet autoimmune conditions. It directly represses Tet2 and hinders DNA demethylation at the FOXP3 CNS2 region. This event weakens the differentiation and stability of regulatory T cells. miR-142-3p also targets Stat5, Smad3 and TGFβ receptors. These signals jointly disturb regulatory T cell homeostasis. Blocking miR-142-3p restores Tet2 expression and rescues defective regulatory T cell function [36,37,38,39]. This strategy offers therapeutic potential for islet autoimmunity and type 1 diabetes. These observations are derived from immune cell experiments and NOD mouse models. Upregulated miR-92a drives islet autoimmunity onset. It targets Ctla4, Foxo1, Pten, and Klf2 to activate PI3K signaling. This promotes TFH precursor generation and suppresses naive Treg induction. miR-92a together with miR-181a participates in multiple autoimmune disorders beyond islet autoimmunity [40,41]. This evidence comes from mouse autoimmune disease models.

MiR-146a is a critical anti-inflammatory microRNA involved in multiple diabetes-related inflammatory disorders. Its expression is significantly reduced in peripheral blood mononuclear cells and serum of patients with type 2 diabetes. The serum miR-146a level shows an inverse association with the pro-inflammatory cytokine IL-8 in type 2 diabetes [42,43]. These data are obtained from human patient peripheral blood samples. Functional experiments have validated the protective role of miR-146a against diabetes related inflammation. The upregulation of miR-146a suppresses inflammatory cytokine expression in inflammatory cell models. In vivo studies further confirm that miR-146a alleviates high-fat diet-induced inflammatory damage in adipose tissues [44,45]. These results come from cell culture and high-fat diet-induced insulin resistance mouse models. Several other microRNAs also participate in diabetes development and progression. miR-214 modulates glucose metabolism and regulated cell death to affect diabetes related pathological changes [46,47]. Evidence includes hepatocyte cell experiments and diabetic mouse models. miR-147 mediates macrophage inflammatory responses, which contribute to systemic insulin resistance in diabetic conditions [48,49,50]. Its functional role remains controversial and needs further clinical verification. Conflicting results exist between mouse model experiments and limited human sample data. MiR-126 protects against high glucose-triggered inflammation by promoting anti-inflammatory cytokine secretion [51]. This conclusion derives from human gingival fibroblast cell experiments.

Distinct miRNA sets regulate autoimmune beta-cell injury in type 1 diabetes. Other miRNAs control insulin resistance in adipose liver and skeletal muscle for type 2 diabetes. A small subset of miRNAs carry conflicting functional conclusions. More human islet and insulin resistance patient sample studies are needed to resolve these inconsistencies.

## 5. miRNAs and Tumors

Aberrant miRNA expression is widely detected in human cancers. MiRNAs regulate oncogenes and tumor suppressor genes. They affect tumor onset development invasion and metastasis. They serve as candidate biomarkers for cancer diagnosis treatment and prognosis assessment, as shown in Figure 2 and Table 1. We organized this section according to tumor types and miRNA functional classification. We separate oncogenic miRNAs and tumor suppressive miRNAs for each tumor subtype.

MiR-21 acts as a typical oncogenic miRNA. It presents high expression in many cancer types. It inhibits tumor suppressor molecules, including PTEN PDCD4 and TPM1. It also regulates FOXO3a phosphorylation through the PI3K/AKT pathway. It promotes breast cancer progression [52]. This mechanism is validated in breast cancer cell lines and xenograft mouse models. Reduced miR-21 expression can restrain the invasion and metastasis of non-small-cell lung cancer cells [52]. Evidence comes from lung cancer cell experiments. Circulating miR-21 levels rise in patients with pancreatic cancer and lung cancer. It relates to clinical prognosis and drug resistance. It also shows high expression in non-smoking patients with lung cancer with EGFR mutation. Blocking miR-21 can improve cancer cell sensitivity to EGFR tyrosine kinase inhibitors [52]. These observations are supported by human patient clinical sample data and cell experiments.

Apart from miR-21, multiple miRNAs also participate in tumor regulation. MiR-29 family members show low expression in liver cancer and breast cancer. They target CDK6 and KLF4. They regulate DNA methyltransferase. These miRNAs activate FHIT and WWOX tumor suppressor pathways. They exert anti-tumor effects [53,54,55]. These results are validated in liver and breast cancer cell lines and xenograft animal models.

Let-7 belongs to the earliest identified miRNA family. Its expression decreases in various tumors. It mainly targets HMGA2 and RAS. Changing let-7 levels can alter the proliferative activity of tumor cells. In vivo delivery of let-7 can hinder lung cancer progression in animal models [56,57,58,59]. Genetic variation in KRAS 3′UTR disturbs the binding of let-7. Such variation closely links to clinical prognosis of lung cancer and breast cancer [56,57,58,59]. These data come from lung cancer mouse models and human patient cohort analyses.

For prostate cancer, MiR-145 modulates multiple oncogenes. It regulates diverse cellular processes. It participates in disease transition from localized state to metastatic status [60,61]. This evidence includes urinary extracellular vesicle detection from patients with prostate cancer and prostate cancer cell experiments. The MiR-148-3 family shows downregulation in high-grade tumors. It may function as a tumor suppressor in prostate cancer. It acts as a potential biomarker for disease detection [62,63]. This conclusion is supported by human prostate tumor tissue samples. The functional regulatory role of miR-185 in prostate cancer still remains unclear. Some reports show tumor-suppressive effects. Other groups obtain neutral or opposite experimental results [64,65]. These contradictory findings remind readers to interpret existing data with caution.

In hepatocellular carcinoma, highly expressed miR-21 and miR-221 aggravate malignant progression. They target the PTEN suppressor network [66]. The JAK-STAT pathway gets abnormally activated under the regulation of disordered miRNAs. Upregulated miR-155 inhibits the expression of key tumor suppressor SOCS1 [67,68]. Downregulated miR-637 weakens its inhibitory effect on STAT proteins [69,70]. These mechanisms are obtained from liver cancer cell experiments and human hepatocellular carcinoma tissue samples.

Oncogenic and tumor-suppressive miRNAs exert context-dependent functions across different tumor types. Many conclusions are derived from cell lines and xenograft animal models. Some miRNAs display conflicting functional results from different research groups. Circulating miRNAs show promising diagnostic value. Large multi-center clinical cohorts are required for further validation.

## 6. miRNAs and Cardiovascular Diseases

### 6.1. miRNAs and Heart Failure

Heart failure constitutes a severe clinical manifestation or end-stage of multiple cardiac disorders, with pathological cardiac remodeling as a core feature. miRNAs contribute to heart failure development and progression by regulating cardiomyocyte hypertrophy, apoptosis, and fibrosis. Prior research has verified that miR-1254 and miR-1306-5p are markedly positively associated with mortality risk among patients with heart failure. An aberrant overexpression of miR-132 drives pathological cardiac remodeling, promoting heart failure onset and progression. CDR132L, a synthetic antisense oligonucleotide targeting miR-132, reverses cardiomyocyte hypertrophy, restores autophagic and calcium signaling homeostasis, and alleviates myocardial fibrosis [71,72,73,74]. Preclinical evidence is obtained from transverse aortic constriction mouse models and large-animal heart failure models. Phase 1b clinical trial results have also been reported for this drug candidate.

MiR-208a is specifically expressed in cardiomyocytes. It exerts vital effects on cardiac remodeling and heart failure development. Suppressing miR-208a can halt pathological cardiac remodeling. In rats with diastolic heart failure, antimiR-208a relieves cardiac damage and functional decline in a dose-dependent manner. It also regulates myosin isoform transformation and elevates survival rates [75,76]. This result derives from rat heart failure animal models. MiR-652 expression obviously rises in mice with transverse aortic constriction. LNA-antimiR-652 can restore cardiac function. It alleviates fibrosis, inhibits cell apoptosis, lowers BNP level, and facilitates angiogenesis. Jagged1 is confirmed as its direct target gene [77]. Evidence comes from mouse pressure overload heart failure models. Recent studies prove that miR-27b-3p is a core regulator of cardiac remodeling. Silencing miR-27b-3p targets FGF1 to elevate PGC-1α/β expression. It strengthens mitochondrial oxidative phosphorylation and restrains cardiomyocyte hypertrophy. Thus, miR-27b-3p is a promising therapeutic target for remodeling-related cardiovascular disorders [78]. This conclusion is supported by cardiomyocyte cell experiments and mouse cardiac remodeling models.

Notably several human cohort studies yield inconsistent observations for some above-mentioned heart failure-related circulating miRNAs. Certain candidate circulating miRNAs show good prognostic performance in small-scale patient cohorts yet fail to deliver equivalent predictive power in independent larger patient populations. Such discordance may arise from varied patient baseline characteristics including underlying comorbidities and medication history. These observations highlight the necessity for the cautious interpretation of preclinical findings before clinical translation.

### 6.2. miRNAs and Myocardial Infarction

Myocardial infarction is a critical cardiovascular emergency. Insufficient blood supply triggers myocardial ischemia, hypoxia, and tissue necrosis. MiRNAs participate in their pathological progression, tissue repair, and prognostic evaluation. Multiple miRNAs control cardiomyocyte death inflammatory response and post-infarction ventricular remodeling. Most functional studies use myocardial infarction rodent models. Circulating miRNAs in plasma can reflect myocardial injury in human patients. MiR-146a can predict poor clinical outcomes of myocardial infarction. This finding is supported by human patient plasma sample analysis. Suppressing miR-222 regulates the PI3K/AKT pathway to relieve myocardial damage. Evidence comes from myocardial infarction mouse models. As part of the miR-17-92 cluster, miR-92a restricts angiogenesis. Blocking miR-92a facilitates vascular growth, optimizes ventricular function, and shrinks the infarct area, thus protecting the heart [79,80,81,82,83,84]. Data are collected from mouse large-animal ischemia–reperfusion models and early-phase human clinical studies. The miR-34 family is a promising therapeutic target for myocardial infarction. Its suppression relieves post-infarction cardiac remodeling and dysfunction. It also ameliorates pathological cardiac hypertrophy without raising cancer risks, showing good application value in treating myocardial infarction and heart failure [85,86]. These results are from mouse myocardial infarction models. Multiple miRNAs are confirmed as effective therapeutic targets in preclinical research. Inhibiting miR-15 lessens infarct size and ameliorates cardiac function in a dose-dependent manner [87]. This observation comes from mouse myocardial infarction models. MiR-22 suppresses autophagy; its inhibition activates autophagy and delays cardiac remodeling in aged mice [88]. Evidence derives from aged mouse myocardial infarction models. Upregulating miR-99a inhibits hypoxia-induced cell apoptosis via the mTOR/P70S6K pathway and improves left ventricular function and survival rate [89]. This conclusion is supported by cell hypoxia experiments and mouse myocardial infarction models.

Delivering miR-214 via adenovirus improves ventricular remodeling and reduces cardiomyocyte apoptosis [90]. This result comes from rat myocardial infarction models. MiR-133 mediates the anti-apoptotic and cardioprotective effects of carvedilol under oxidative stress [91]. This finding is obtained from cardiomyocyte oxidative stress cell experiments. MiR-210 regulates mitochondrial metabolism and alleviates myocardial ischemia–reperfusion injury [92]. Evidence includes cell and mouse ischemia–reperfusion models.

A large panel of miRNAs regulate cardiomyocyte survival angiogenesis and ventricular remodeling after myocardial infarction. Several miRNA-targeting molecules have entered early-phase clinical trials. Circulating miRNAs serve as candidate prognostic markers for human myocardial infarction patients.

A small subset of miRNA-targeting interventions produce variable outcomes across different preclinical experimental settings. Some protective effects observed in young rodent models cannot be fully reproduced in aged animal subjects that better mimic real-world patient demographics. Circulating miRNA biomarkers for myocardial infarction also display inconsistent fold change magnitudes across separate clinical cohorts. Standardized multi-center validation work remains essential.

### 6.3. miRNAs and Cardiac Fibrosis

Cardiac fibrosis frequently occurs during the progression of cardiovascular diseases toward heart failure. It is mainly featured by excessive interstitial collagen accumulation. MiRNAs regulate cardiac fibrosis through modulating related signaling cascades. MiR-21 and miR-208b activate the TGF-β1/Smad3 pathway to accelerate post-infarction cardiac fibrosis. MiR-21 is highly expressed in cardiac fibroblasts of failing hearts. AntagomiR-mediated miR-21 knockdown lowers ERK-MAPK activity. It further restrains interstitial fibrosis and restores cardiac function. In angiotensin II-triggered myocardial fibrosis, osteopontin upregulates miR-21. It targets the PTEN/Smad7 axis to maintain fibroblast viability [93,94]. These results are validated in cardiac fibroblast cell experiments and mouse cardiac fibrosis models. The miR-29 family is a key anti-fibrotic miRNA family widely implicated in fibrosis across multiple organs, including the heart, liver, and lungs, and suppresses cardiac fibrosis via the TGF-β/Smad pathway [95,96,97]. Evidence comes from multiple organ fibrosis animal models.

Limited human tissue data exist to corroborate many anti-fibrotic miRNA effects identified in animal systems. A few human cardiac tissue studies report divergent expression trends for miR-29 family members under fibrotic pathological states. Such discrepancies warn against the direct extrapolation of animal-derived anti-fibrotic miRNA conclusions to human cardiac fibrosis.

### 6.4. miRNAs and Arrhythmia

Arrhythmia refers to disturbances in cardiac electrical activity, including abnormalities in rate, rhythm, origin, conduction velocity, or excitation sequence. miRNAs regulate ion channel gene expression, modulate cardiac excitability, and contribute to arrhythmogenesis. The muscle-specific miR-1 regulates ion channel expression and cardiac excitability: Yang et al. found that miR-1 inhibits connexin 43 and the potassium channel subunit Kir2.1, slowing conduction in ischemic myocardium, reducing membrane potential, and promoting ischemic arrhythmias. Another study showed that miR-1 overexpression targets laminin receptor 2 phosphorylation, enhances Ca^2+^ release, and promotes arrhythmogenesis. LNA-antimiR-1 prevents or reverses these pathological changes, identifying miR-1 as a potential therapeutic target [98,99,100,101]. These conclusions derive from cardiomyocyte cell experiments and rodent ischemia arrhythmia models. miR-26 is a cardiac-enriched miRNA whose expression is downregulated in atrial fibrillation animal models and patient atrial tissues. Endogenous miR-26 knockout promotes atrial fibrillation in mice, while miR-26 overexpression reduces arrhythmia susceptibility [102]. Evidence includes human patient atrial tissue samples and mouse atrial fibrillation models.

Some ion channel-regulating miRNAs exert context-dependent functions. Their pro-arrhythmic or anti-arrhythmic properties may shift depending on underlying cardiac disease background. Several candidate arrhythmia-related miRNAs show inconsistent expression patterns when compared between atrial fibrillation patient atrial tissue and peripheral circulating biofluids.

### 6.5. miRNAs and Atherosclerosis

Atherosclerosis is a chronic vascular disease. Its main features include endothelial damage, lipid deposition, and inflammatory response. It acts as the key pathological foundation for coronary heart disease and cerebral infarction. MiRNAs control endothelial function macrophage lipid metabolism and vascular inflammation during atherosclerosis progression. Most functional evidence comes from ApoE^−^/^−^ mouse atherosclerosis models. Human plasma samples detect expression shifts of multiple circulating miRNAs from patients with atherosclerosis. MiRNAs exert regulatory effects throughout the development of atherosclerosis. Studies by Sun et al. confirmed decreased miR-181b levels. Such a reduction was found in the aortic intima of high-fat diet ApoE^−^/^−^ mice and the plasma of patients with coronary artery disease. Injecting liposome-wrapped miR-181b mimics suppresses vascular NF-κB activity. It also inhibits pro-inflammatory gene expression and alleviates atherosclerotic plaque formation [103]. This result comes from ApoE^−^/^−^ mouse model and human patient plasma samples. miR-29 contributes to arterial integrity; Ulrich et al. demonstrated in atherosclerosis mouse models that long-term subcutaneous injection of LNA-antimiR-29 reduces lesion size and enhances plaque stability, providing a novel non-lipid-lowering therapeutic strategy [104,105]. Evidence is obtained from ApoE^−^/^−^ mouse atherosclerosis models. miR-33 regulates macrophage cholesterol efflux; the inhibition or macrophage-specific deletion of miR-33 reduces lipid accumulation and inflammation, thereby decreasing plaque formation [106]. This conclusion derives from macrophage experiments and ApoE^−^/^−^ mouse models. miR-302a promotes macrophage cholesterol excretion and inhibits foam cell formation. Furthermore, miR-126-5p enhances endothelial cell proliferation to exert anti-atherosclerotic effects, while miR-126 modulates pro-inflammatory factor expression and monocyte recruitment into foam cells [107]. These observations come from endothelial and macrophage cell experiments together with atherosclerosis mouse models.

MiRNAs regulate endothelial injury macrophage lipid handling and vascular inflammation in atherosclerosis. Preclinical animal data support their therapeutic potential. Circulating miRNAs serve as candidate plasma biomarkers for human atherosclerotic vascular disease.

A small number of preclinical investigations report neutral or even adverse vascular outcomes for certain miRNA interventions that otherwise display anti-atherosclerotic activity under distinct experimental conditions. Circulating miRNA biomarker performance for atherosclerosis varies heavily across patient cohorts with different cardiovascular risk factor burdens.

## 7. miRNAs and Kidney Diseases

The pathogenesis of kidney diseases is closely associated with renal tissue injury, inflammatory responses, and cellular apoptosis. miRNAs participate in kidney disease onset and progression by regulating relevant target genes, as shown in Table 1. Previous studies showed that miR-377 promotes glomerular fibronectin deposition, which may contribute to renal injury [108]. This finding is validated in diabetic nephropathy cell and animal models. Plenty of evidence shows miR-21 suppresses PDCD4 and PTEN. It can protect the kidneys against ischemic acute renal injury. In contrast, miR-21 knockdown triggers renal tubular cell apoptosis and worsens kidney damage [109]. Evidence comes from renal tubular cell experiments and mouse renal–ischemia–reperfusion models. Elevated miR-668 restrains MTP18 expression. This effect relieves hypoxia-caused mitochondrial damage. Low miR-668 expression aggravates such mitochondrial lesions [110]. This conclusion derives from in vitro hypoxia cell experiments and mouse acute kidney injury models. Increased miR-34a regulates ATG4B expression. It boosts autophagy in renal tubular epithelial cells. Ultimately, it aggravates the progression of acute kidney injury [111,112]. These results are supported by renal cell culture and mouse acute kidney injury models.

A proportion of existing kidney-focused miRNA studies deliver conflicting functional conclusions. For instance miR-21 exhibits context-dependent activities within renal compartments. It confers protective effects during acute ischemic kidney injury yet can drive fibrotic pathological changes in chronic progressive nephropathy. Several miRNA protective phenotypes observed in rodent acute kidney injury models cannot be consistently replicated in limited available human renal biopsy specimens. Some candidate circulating miRNA markers for kidney injury also show inconsistent expression trends across separate patient cohorts. Such inconsistencies may relate to differences in renal injury etiology patient age and sample collection timing.

MiRNAs participate in renal inflammation mitochondrial homeostasis autophagy and fibrotic remodeling across multiple kidney disease subtypes. The majority of mechanistic evidence originates from cell culture and rodent animal models. Human tissue and large patient cohort datasets remain comparatively limited. Further research work is required to resolve conflicting experimental observations and validate the clinical utility of candidate miRNA biomarkers and therapeutic targets for human kidney disorders.

Table 1 shows representative miRNAs target genes and pathological outcomes across major human diseases. This table summarizes key miRNA molecules and their downstream target genes or signaling pathways and corresponding pathological outcomes in central nervous system diseases, diabetes, malignant tumors, cardiovascular diseases, and kidney diseases.

## 8. Discussion

MicroRNAs participate in pathological regulation across multiple human organ systems. They modulate core biological processes such as inflammation and cell apoptosis. They also control cell proliferation, fibrosis, and neuronal remodeling. The same microRNA can exert distinct even opposite functions in different tissue microenvironments. Take miR-21 as an example. In tumor tissues, miR-21 acts as oncogenic miRNA. It inhibits multiple tumor suppressor genes such as PTEN and PDCD4. It facilitates cancer cell proliferation invasion and metastasis. In cardiac fibroblasts, miR-21 activates ERK-MAPK signaling. It promotes myocardial interstitial fibrosis after myocardial infarction. Nevertheless miR-21 produces protective effects in acute renal ischemic injury. It reduces renal tubular cell apoptosis. Such tissue-specific dual-function property brings big challenges for systemic miRNA-targeted therapy. Tissue-selective delivery strategies become necessary to balance therapeutic efficacy and safety risks.

Most existing research data are obtained from rodent disease models. Relevant results have laid solid basic foundations for subsequent translational study. Growing large animal experiments and human sample analyses further verify miRNA functions. Synthetic microRNA mimics and antisense oligonucleotides show prominent therapeutic value in preclinical studies. Researchers have developed multiple mature solutions to overcome delivery-related challenges. Novel biomaterial carriers effectively prevent free microRNA degradation by nucleases in vivo. Modified oligonucleotide formulations can readily cross the blood–brain barrier. Such technical progress expands miRNA treatment prospects for central nervous system disorders. Exosomes represent one promising delivery platform for therapeutic miRNA. Exosomes possess low immunogenicity and good biocompatibility. They can transfer loaded miRNA molecules to recipient cells. Exosome-mediated miRNA delivery has been tested in preclinical models for neurological tumors and cardiovascular diseases. Still many obstacles remain. These obstacles include large-scale production quality control and tissue-targeting modification of exosome preparations.

Most published reviews only discuss microRNAs within a single disease system. This work summarizes microRNA regulatory profiles in five major disease categories. It identifies shared signaling pathways among neurological, metabolic, cardiovascular, tumor, and kidney diseases. The cross-system comparison provides abundant candidate targets for therapeutic development. It also guides researchers to develop universal miRNA intervention strategies for multiple disorders.

Extracellular miRNAs show tissue-specific expression patterns and high stability in various body fluids. These features make them ideal minimally invasive biomarkers for disease diagnosis and prognosis. Circulating miRNAs can be isolated from plasma, cerebrospinal fluid, and urinary exosomes. Their expression changes reflect pathological damage in multiple organs. These organs include the central nervous system, pancreas, tumor tissue, myocardium, and kidney tissue. The detection of circulating miRNAs faces notable technical limitations. Stem–loop RT-PCR suffers from variation in reverse transcription efficiency across different miRNA sequences. Next-generation sequencing brings batch-effect bias and requires complex data normalization. These technical factors can cause inconsistent detection results across different laboratories. For instance, circulating miR-134 serves as a potential biomarker for epilepsy, as mentioned in Section 3.1. Plasma miR-126 relates to stroke vascular repair described in Section 3.2. Serum miR-21 acts as circulating marker for multiple tumors, as described in Section 5. These candidate markers still need large-sample multi-center prospective cohorts for final validation.

Future translational research needs large prospective cohorts across multiple medical centers. Researchers need to screen and validate panels containing multiple miRNAs. Such multi-marker panels can raise diagnostic sensitivity and specificity. They also avoid unstable test results caused by single miRNA detection. Uniform operating standards are needed for sample collection, preservation, and RNA extraction. These standardized procedures can reduce interference factors. Common interference factors include age, concurrent medication, and combined complications. This work will support the wide clinical use of miRNA early screening tools. These screening tools apply to stroke, neurodegenerative diseases, malignant tumors, heart failure, and diabetic nephropathy.

## 9. Conclusions

MicroRNAs serve as key post-transcriptional regulators across multiple human diseases. Dysregulated microRNA expression triggers pathological damage in diverse organs. Unique microRNA expression patterns can act as sensitive diagnostic molecular markers. MicroRNA-targeted interventions produce remarkable therapeutic outcomes in preclinical research. Continuous advances in carrier technology largely resolve traditional delivery limitations. Optimized delivery systems enable stable and tissue-specific miRNA intervention in vivo. Future research will further refine miRNA-targeted treatment schemes for various illnesses. Sufficient preclinical evidence supports the great clinical transformation potential of miRNA therapy.

## Figures and Tables

**Table 1:** Representative miRNAs across major human diseases.

Disease System	Key miRNA	Target Gene/Pathway
CNS Diseases	miR-134, miR-200b.c, miR-124, miR-133b	LimK1, Aβpathway, TLR4, ERK1/2-STAT3-CREB
Diabetes Mellitus	miR-7, miR-146a, miR-181a	Pax6, NF-xB-IL-8, PTEN-PI3K-NFAT5
Tumors	miR-21, let-7, miR-29	PTEN-PDCD4, HMGA2-KRAS, CDK6-KLF4
Cardiovascular Diseases	miR-132, miR-208a, miR-29, miR-181b	autophagy calcium signaling, myosin isoform transformation, TGF-B1-Smad3, NF-KB
Kidney Diseases	miR-21, miR-377, miR-34a, miR-668	PDCD4-PTEN, fibronectin, autophagy, MTP18

## Data Availability

No new data were created or analyzed in this study. Data sharing is not applicable to this article.

## Abbreviations

The following abbreviations are used in this manuscript:

3′UTR	3′ untranslated region
mRNAs	messenger RNAs
RISC	RNA-induced silencing complex
NF-κB	nuclear factor-κB
TLR4	Toll-like receptor 4
GFAP	glial fibrillary acidic protein
NF200	neurofilament protein 200
GAP-43	growth-related protein 43
GCK	glycolysis by targeting glucokinase
PDHA1	pyruvate dehydrogenase alpha 1
Pax6	paired box gene 6
PDCD4	programmed cell death protein 4
TPM1	tropomyosin 1
CDK6	cyclin-dependent kinase 6
KLF4	Kruppel-like factor 4
HMGA2	high mobility group protein A2
PGC-1α/β	proliferator-activated receptor gamma coactivator 1α/β
FGF1	fibroblast growth factor 1
MTP18	mitochondrial protein 18
ATG4B	autophagy-related gene 4B
